# Potential of SARS-CoV-2 to Cause CNS Infection: Biologic Fundamental and Clinical Experience

**DOI:** 10.3389/fneur.2020.00659

**Published:** 2020-06-18

**Authors:** Jianhan Huang, Meijun Zheng, Xin Tang, Yaxing Chen, Aiping Tong, Liangxue Zhou

**Affiliations:** ^1^Department of Neurosurgery, West China Hospital, West China Medical School, Sichuan University, Chengdu, China; ^2^Department of Otolaryngology, Head and Neck Surgery, West China Hospital, West China Medical School, Sichuan University, Chengdu, China; ^3^State Key Laboratory of Biotherapy, West China Hospital, West China Medical School, Sichuan University, Chengdu, China

**Keywords:** COVID-19, human coronavirus, SARS-CoV-2, central nervous system, viral infection

## Abstract

SARS-CoV-2 is a novel coronavirus leading to serious respiratory disease and is spreading around the world at a raging speed. Recently there is emerging speculations that the central nervous system (CNS) may be involved during SARS-CoV-2 infection, contributing to the respiratory failure. However, the existence of viral replication in CNS has not been confirmed due to the lack of evidence from autopsy specimens. Considering the tropism of SARS-CoV-2, ACE2, is prevailing in CNS, and the neuro-invasive property of human coronavirus was widely reported, there is a need to identified the possible complications during COVID-19 for CNS. In this review, we conduct a detailed summary for the potential of SARS-CoV-2 to infect central nervous system from latest biological fundamental of SARS-CoV-2 to the clinical experience of other human coronaviruses. To confirm the neuro-invasive property of SARS-CoV-2 and the subsequent influence on patients will require further exploration by both virologist and neurologist.

## Introduction

Since the appearance of the first pneumonia patient in early December 2019 in China, the COVID-19 has resulted in a global pandemic. By the end of April 30, over 3,000,000 cases were reported all around the world and leading to over 200,000 deaths (1). Compared with previous coronavirus outbreak, SARS and MERS, COVID-19 has certainly induced a much larger global pandemic. After the application of oxygen therapy, mechanical ventilation, and antiviral therapies, over 90% of the cases have been cured and discharged from hospitals after twice verified absence of virus in respiratory tract by nucleic acid amplification tests (2). Although human coronaviruses mainly lead to respiratory tract infection, previous researches have indeed demonstrated the potential of the coronavirus to spread to extra-pulmonary organs, involving nervous system, gastrointestinal tract, and kidney, as widely observed in SARS and MERS cases. According to the latest reports, some patients with COVID-19 presented neuro-infection symptoms such as olfactory and taste disorders (3), which may be attributed to the olfactory nerve damage. However, the mechanism and route for SARS-CoV-2 to lead to neuro-infection, especially central nervous system (CNS), still remain to be explored. In this review, we summarize the proves for possible SARS-CoV-2 related CNS damage basing on the previous experience of coronavirus infection and the latest outcomes of SARS-CoV-2 biology.

## The Molecular Basis For COVID-19 Neuro-Tropism

Coronavirinae is a group of enveloped, spherical virus in the Coronaviridae family, Nidovirales order, which is further divided into the genus of alphacoronavirus, betacoronavirus, gammacoronavirus, and deltacoronavirus, and were called by a joint name, coronavirus (4). Coronavirus is the largest RNA virus extending to a diameter of 120 nm, with a huge monopartite, linear, single chain, positive RNA genome ranging from 27 to 32 kb inside. The first two strains of coronavirus, namely HCoV-229E and HCoV-OC43, was acquired in 1960's from the organ culture of patients diagnosed with upper respiratory tract disease, and was regarded as a novel kind of pathogen for common cold (5–7). So far, over 3,000 strains of coronavirus are discovered, but most of them prevail in vertebrates such as bats and porcine, and, before the outbreak of COVID-19, only six species of them cause infective disease to human, including HCoV-229E and HCoV-NL63 in alphacoronavirus genus, and HCoV-HKU1, HCoV-OC43, SARS-CoV, and MERS-CoV in betacoronavirus genus (8–12). In most of time, coronavirus infects the epithelium cells of upper respiratory tract, and were regarded as opportunistic pathogens leading to mild infections in immunocompetent individuals, and accounting for about 15% of the common colds all around the world (13). However, the coronavirus can also result in devastating clinical outcomes. Animal coronavirus may sometimes manage to efficient cross-species infection after host-adaptation by novel virus strains and cause zoonotic infections, usually accompanied by lethal cytokine storm. A case in point is the outbreak of pandemic respiratory syndrome caused by SARS-CoV in 2003 and MERS-CoV in 2012. These two strains of coronavirus are no longer restricted in the upper respiratory tract, but also spread to the trachea, bronchi, lung in immunocompetent individuals, and leading to lethal acute respiratory failure. For infants, the aged and patients with disordered immunity, coronavirus sometimes may spread to extra-pulmonary organs, including central nervous system, in which the patients may present high fever and headache with a mortality over 60% (14). In 2019 December, the breakout of zoonotic viral pneumonia revealed a novel kind of human- targeting coronavirus, which was named COVID-19 (15). COVID-19 shares a lot of similarity to the SARS in 2002, both in viral tropism and clinical symptoms, indicating that the COVID-19 may have similar complication with SARS, such as CNS infection. Computer modeling showed the COVID-19 has the same viral receptor as SARS-CoV, which is the angiotensin-converting enzyme 2 (ACE2) (16). The strong binding affinity of spike of SARS-CoV-2 to ACE2 was further proved by biochemical interaction studies and crystal structure analysis (17). As a result, the distribution of ACE2 in tissues plays an important role in SARS-CoV-2 tissue tropism.

### Spike Protein and Viral Tropism

The shape of coronavirus under electron microscopy is quite distinctive, in which the virus was found to be surrounded by a number of projections connected to viral envelope, and make the virus look like a royal crown (18). These projections are called “Spike(S),” which are proteins determining the tropism of coronavirus. The *S* protein is a glycosylated transmembrane protein, consisting of two subunits, S1 and S2, which are separately responsible for the viral attachment and membrane fusion during the virus enter its host cell (19). During the process of viral entry, the virus makes use of the receptor binding domain locating in the distal S1 subunit to attach to the viral receptor on the membrane of host cell. Then the S2 subunit is cleaved by the transmembrane protease, leading to an irreversible conformational change and the subsequent membrane fusion of virus and the host cell (20). As a result, the entry of coronavirus into host cell turns out to be a multistep process, and each of the mutation in the functional domain of S1 or S2 subunit may result in different viral tropism or even infectivity. In the latest research conducted by Vincent Munster et al., the author created various COVID-19 pseudotypes with spikes from different lineage B coronaviruses, some of which infect animals, and revealed that the absence of 431–437 and 456–473 residues in the RNA coding sequence of receptor binding domain, which is widely observed in betacoronaviruses that infect non-human animals, deprived the infectivity of modified COVID-19 to infect cells overexpressed human angiotensin-converting enzyme 2 (hACE2), the receptor for COVID-19 (21). The infectivity recovered after trypsin digestion, indicating these regions are related to the intact function of human transmembrane protease, and act as a major barrier for mutual infections between different species, at least in some of the animal betacoronaviruses. Considering the huge coronavirus reservoir in wild animals may acquire the necessary RNA fragment from human coronavirus by recombination, it is no doubt that zoonotic coronavirus infections will appear at a much higher frequency in the near future without manual intervention (22). Although the viral replication cycle is driven by a series of proteins or organelles in host cell and the absence of each of them can lead to disrupted viral propagation, the entry of the virus to the host cell is the primary step for all of the subsequent process. As a result, using neutralizing antibody or recombinant protein to keep the virus from entry into the host cell is the most common strategy to treat and prevent viral infection. The indispensable function of the *S* protein during viral entry makes it an ideal target for antibody or vaccine development (23). As shown in the case that Weiner et al. produced a plasmid-based MERS-CoV S protein vaccine using 293T cells. Vaccine-immunized mice showed increased T cell number, elevated T cell response, protective antibody, and resistance to MERS-CoV infection (24).

### The Function and Distribution of ACE2

Discovered in 2000 as the first homolog of human angiotensin-converting enzyme, angiotensin-converting enzyme (ACE2) is a transmembrane carboxypeptidase, which removes carboxyterminal amino acid from peptide substrates by peptide hydrolyzation reaction (25). ACE2 shares about 42% similar coding sequence with ACE, and was proposed to be the product of a fusion gene consist of partial ACE and collectrin. As a result, ACE2 turn out to be a multi-functional protein with two separate functional domains. On one hand, physically, as a member of the renin angiotensin system, ACE2 converts angiotensin I to Ang 1-9, a peptide whose function remains to be explored, and angiotensin II to angiotensin 1-7, which is a vasodilator as the ligand for the G-protein coupled receptor MAS1 (26, 27). On the other hand, its collectrin domain collaborates with amino acid transporter B0AT1 to transfer neutral amino acids on the brush border of intestinal epithelial cells, and was found to be essential for the absorbance of several amino acids, especially tryptophan (28, 29). According to the immunohistochemistry, the ACE2 mainly distributes in the vascular endothelial cells of the heart, kidney, testes, alveoli, gastrointestinal, and, at a lower expression level, brain (30–34). The ACE2 in brain was also found in the neurons of subfornical organ, an area lacking the blood-brain barrier and sensitive to blood-borne circulating peptides (35).

The recognition of the existence of ACE2 in brain is much earlier than the identification of ACE2 molecule. In 1988, Santos et al. observed a continued transformation of ANG-I to Ang-(1–7) even after using ACE inhibitor in the brainstem of dog, which indicated the Ang-(1–7) was synthesized via a different route bypass ACE, which was later confirmed to be ACE2 pathway (36). Soon the same group verified that Ang-(1–7) produced by this ACE homolog in hypothalamo-neurohypophysial system (HNS) induced the release of arginine vasopressin (AVP) when Ang-(1–7) or angiotensin II (Ang II) was added to the explants from rats (37). One year later, Campagnole-Santos et al. provided the first *in vivo* proof for the biological functions of central Ang-(1–7) (38). After the injection of Ang-(1–7) into the nucleus tractus solitarii (NTS), the rats present a significant reduction in blood pressure. The function of Ang-(1–7), the products of ACE2, in central nervous system was further confirmed to be of great importance in baroreflex modulation and the central control of the blood pressure by the Ang-(1–7) antagonist, its analog D-Ala7-ANG-(1–7) (39–41). In spontaneously hypertensive rat models, selectively overexpressing ACE2 in the rostral ventrolateral medulla or paraventricular nucleus induced a significant relief on blood pressure (42, 43). Besides, the ACE2 and the renin-angiotensin system (RAS) also play an important role in neuro-inflammation. Zheng et al. used triple transgenic mice selectively overexpressing ACE2 in neurons, SARA, to study the role of ACE2 in ischemic stroke (44). After *in vitro* deprivation of oxygen and glucose for the brain slices from transgenic mice and control mice, they found less swelling, cell death, and ROS production in cerebral cortex and hippocampal CA1 region areas. The latest research also revealed that the ACE2 in brain not only responsible for the body blood pressure regulation, but also present a protective profile for the brain itself in a series of neurologic pathologies, including aging-related neuroinflammation (45), focal cerebral ischemia (46), demyelinating disease (47), Alzheimer's disease (48), and neuropsychiatric disorders (49). In general, the receptor of SARS-CoV-2, ACE2, is widely distributed in the central nervous system, and has been proved to take part in multiple normal physiological processes. As a result, once SARS-CoV-2 successfully invades central nervous system, it can infect neurons by recognition of ACE2, and then leads to central nervous system damage via direct viral replication or disordered immune response.

## The Possible Routes For COVID-19 Infection From Respiratory Tract to Brain

**Figure 1:**
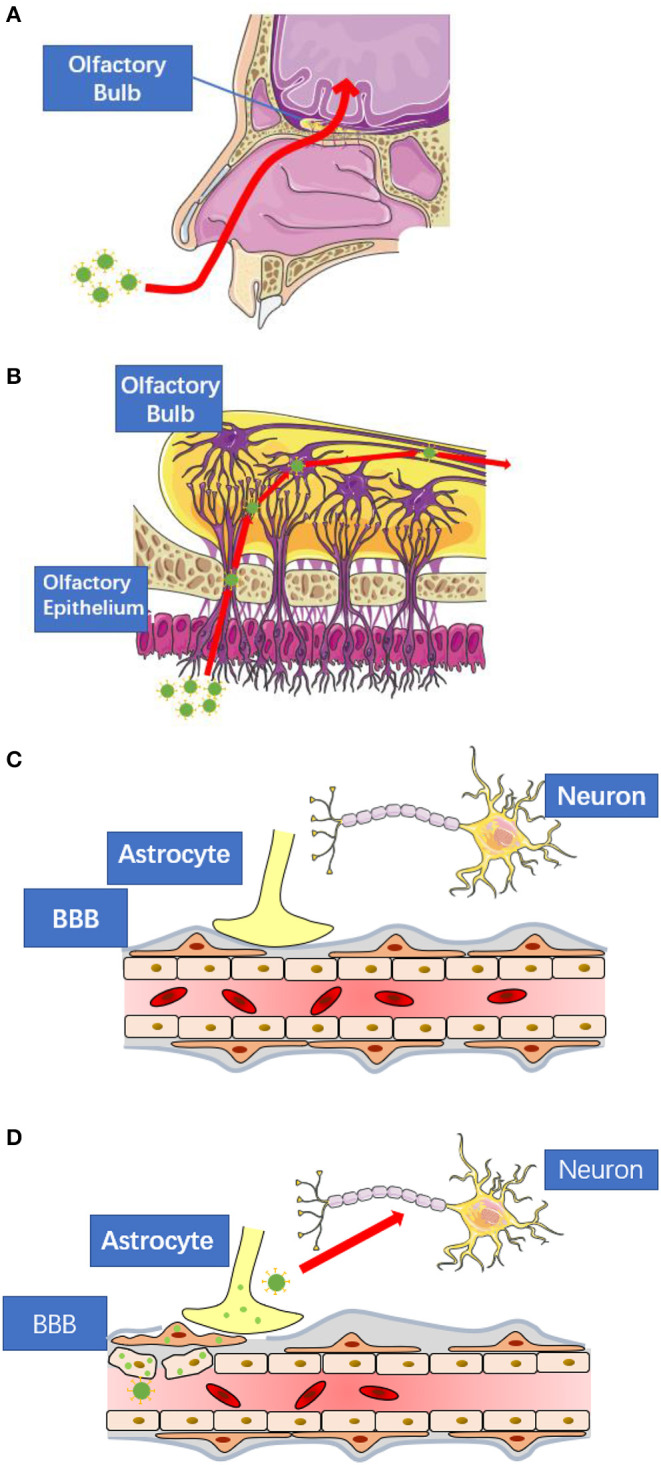
The possible routes for SARS-CoV-2 to enter central nervous system. **(A)** SARS-CoV-2 may spread to the central nervous system via retrograde transport through the olfactory nerve as seen in the case of other respiratory viruses. **(B)** The nerve endings of olfactory receptor neurons distribute in the nasal mucosa. These neurons are exposed to SARS-CoV-2 and are connected to the olfactory bulb. **(C)** The blood-brain-barrier consists of tightly connected endothelial cells, basement membrane, and the end feet of astrocytes. All of them prevent large molecules to enter the central nervous system directly. **(D)** When viral infection occurs in the endothelial cells, pericytes, or astrocytes, the blood-brain-barrier will lose its protective function. These figures are modified and reproduced under the permission of Creative Commons Attribution 3.0 License.

The cases of respiratory virus induced CNS infection have been widely reported, such as adenovirus, influenza virus and measle virus. As another respiratory virus, SARS-CoV-2 also has the potential to enter the CNS via retrograde transport or circulatory system. In this section, we introduce the common pathway, retrograde transport and circulatory route, for respiratory virus-induced CNS infection, in which there may be clues for clinical researchers to find viral replication (Figure 1).

### Retrograde Transport Through Olfactory Nerve

Neurologic viral infection is very common, but most of the neural infections remain in peripheral nervous system and do not lead to serious damage to healthy individuals. It is estimated that about 70–80% of healthy adults are infected by human simplex virus (HSV) including HSV-1 and HSV-2, both of which lead latent but lifelong infection in the cell bodies of neurons, such as trigeminal ganglion. However, when virus enter the central nervous system through neuronal dissemination, which is named retrograde transport, it may induce life-threatening diseases, such as herpes simplex encephalitis, which has a rate of fatality as high as 70% in untreated patients (50). Other than HSV, many viruses can take use of the retrograde transport to enter the central nervous system, such as influenza virus (51), measle virus (52), vesicular stomatitis virus (53), and rabies (54). These viruses may first infect the local tissue, and then spread to the peripheral nerves by binding to the specific viral receptor on the axons or dendrites of the neurons. Once the viruses enter the neurons, they reside in the endosomal vesicles, which is formed by the cytomembrane during viral entry, and engage a motor protein, dynein, to transport the endosomal vesicles along the microtubule to the centrosome locating beside the nucleus (55). The capsid of virus gradually disassembles according to the change of the gradually decreasing PH value in the endosomal vesicle, and then the nucleic acids of the virus are released from endosomal vesicle to the cytoplasm (56). After that, most of the DNA virus will dock into the nucleus, where there are transcriptase and substrates for mRNA and DNA synthesis, while most of the RNA virus stay in the cytoplasm and start to form the inclusion body, where RNA virus replicates. Finally, viral nucleic acids and viral protein are transported to synaptic membrane for further assembly and trans-synapse transmission to the next neuron and ultimately to the central nervous system.

Given the nerve endings distribute in all types of tissues, and the constitutions of different nerve pathways are not the same, the retrograde transport in specific disease can undergo different transport process and lead to varied clinical features according to the location of the primary lesion. Since SARS-CoV-2 distributes in the respiratory tracts, the olfactory nerve may servesas a major retrograde route for the spread of virus to central nervous system, which has been widely reported in vesicular stomatitis virus and influenza virus (57–59). During the transduction of odor signal, the stimulation starts from the dendrites of the OSN, which scatter under the olfactory mucosa on the roof of the nasal cavity (60), then transmits along the axon of the neuron, which combined together into bundles, to the central olfactory apparatus, including olfactory bulb and rhinencephalon (61). To confirm the SARS-CoV-2 related CNS infection, the viral replication in historical specimens of these location should be paid most attention.

### Viral Spread Through Circulatory System

Another possible pathway for COVID-19 is to enter the CNS through the circulatory system, in which the involvement of CNS acts as part of the systematic infection derived from the primary lesion. This pathway starts from the release of the virus from the basolateral side of the infected epithelial cells. Different from the systematic spread of viral infection commonly seen in severe infection cases to marrow or kidney, due to the protection of blood-brain barrier, neurons are not in direct contact with circulatory system. Instead, virus take use of two types of intermediate host cell before spreading into CNS from circulatory system, namely the endothelium and leukocytes (62).

The blood-brain barrier (BBB) is a highly selective structure separating CNS from the circulating blood to avoid most of the large molecules entering CNS, including virus. This barrier consists of three layers (63). The first layer is formed by endothelial cells lining around the brain capillaries, which has a distinct tight junction connection different from endothelium in other tissue. The second layer is a thick, intact basement membrane, and finally the astrocyte end-feet projections surrounding the capillaries forms the third layer (64, 65). During viremia, some viruses can directly infect the epithelial cells of blood-brain barrier, which is of the most exposure to the circulating virions among the three layers. The viral replication and the subsequent inflammation cytokines eventually deprive BBB of the normal function by increasing its permeability, and lead to the CNS infection. This pathway is confirmed in the cases of Encephalitic Alphaviruses (66), Hepatitis E Virus (67), and HSV (68). Considering the viral receptor, ACE2, is highly expressed in the epithelial cells, it is possible for COVID-19 to cause direct damage to the BBB, and thus enter the CNS.

Instead of penetrating the BBB, another group of viruses, including human immunodeficiency virus (69) and Zika virus (70), take use of the circulating leukocytes to carry them across the BBB, which is called “Trojan horse” mechanism (71). This pathway is best explored in the case of AIDS-related dementia (72). During HIV infection, the virus accumulates in the monocytes, which usually do not undergo a rapid replication-induced cytolysis and are able to traverse the BBB physically (73). After entering the CNS, HIV infects and activates the microglia to secret chemokines, which in turn induce a larger size of monocytes infiltration, and increase the permeability of BBB as widely seen in the normal process of inflammation (74, 75). The ability to infect leukocytes is also reported in coronavirus infection. For example, HCoV-229E can lead to restricted infection in monocytes/macrophages (76, 77), peritoneal macrophages and dendritic cells (78–80), and induce chemokine secretion (81), which means coronavirus has the same potential as HIV to constitute a reservoir in leukocytes and use them as vectors to spread into the other tissues outside the respiratory tracts.

## The Neuro-Invasive Property of Coronavirus

Although traditionally coronaviruses were regarded as respiratory viruses for a long time, there are indeed accumulating proves about the neuro-invasive property of coronavirus these years. Among the six strains of coronaviruses before the breakout of COVID-19, three of them, HCoV-229E, HCoV-OC43, and SARS-CoV, have ever been reported to cause CNS infection so far. Given the prevalent existence of the coronavirus in healthy individuals and the lethal outcomes of the central nervous system infection, increasing efforts are devoted into the research of coronavirus neurological pathologies. In this section, we summarize the previous researches on coronavirus CNS infection, including clinical cases, histopathology, and animal models, and mainly focus on the SARS-CoV. Since SARS-CoV shares the same viral receptor as SARS-CoV-2 and also induced a serious pandemic lethal pneumonia, we suppose SARS-CoV-2 can also lead to CNS infection with similar mechanism observed in SARS cases.

### The Neuro-Invasive Property of Murine Hepatitis Virus (MHV)

Actually, some animal-oriented coronaviruses have long been regarded as neuro-invasive viruses and were used in the research of virus-related neurological disease. A case in point is murine hepatitis virus (MHV) is a highly infectious coronavirus, which was discovered and isolated in 1949 and prevailing in many mouse colonies throughout the world (82). These zoonotic pathogens are mainly transmitted via respiratory route, and lead to hepatitis and encephalitis. After intracerebral injection of the MHV, the rats presented acute panencephalitis and demyelinating foci, and the virus RNA was detected in both neurons and oligodendroglial cells (83). This discovery in animal models reminded people that this respiratory virus may also have the potential to induce central nervous system, but the routes were still remained unclear until 1988 when the olfactory neural pathway was confirmed to be the routes for CNS infection caused by respiratory virus instead of trigeminal nerve pathway (84, 85). Later it was found that MHV has a strong correlation with the autoimmune neurogenic inflammation and the subsequent multiple sclerosis, indicated that the coronavirus may result neurologic disorder via distorted immune attack other than direct viral replication (86). Nowadays, MHV has been widely used in the construction of animal models of multiple sclerosis or neurological infections to study the functional change and mechanism of neurological diseases (87–89).

### Neuro-Invasive Property of HCoV-229E and HCoV-OC43

HCoV-229E and HCoV-OC43 are the first two strains of coronaviruses been isolated. In most of the time these two coronaviruses induce mild infection confined in upper respiratory tracts. Since the discovery of the neuro-invasive property of the MHV in mice, some researchers supposed human coronaviruses may also lead to human neurological disease, especially multiple sclerosis. In 1981, two strains of coronaviruses which serologically related to HCoV-OC43 and murine coronavirus A59 were isolated from the fresh autopsy brain tissue of patients diagnosed with multiple sclerosis (90). These two strains of coronaviruses shared cross-reactivity to the OC43 antiserum, but due to the lack of polymerase chain reaction (PCR) technology, these two viruses could not be clearly identified. Until 1992, Stewart JN et al. confirmed the existence of HCoV-OC43 and HCoV-229E in the brain tissue of multiple sclerosis patients by total RNA extracting and reverse transcription-polymerase chain reaction, in which the HCoV-OC43 but not HCoV-229E was found in specimens of all the patients (91). Actually, HCoV-229E and HCoV-OC43 present a different infectivity to the cells of the central nervous system. Both of the two coronaviruses can lead to acute infections to the cell lines of neuroblastoma, glioblastoma, glioblastoma, astrocytoma, and oligodendrocytes (92), but only HCoV-OC43 can result in a persistent infection (92). Nowadays, HCoV-OC43 is considered to be related to a series of chronic neurological disorders such as medullary atrophy, Parkinson's disease, polyneuropathy, senile dementia, and headache (93). According to the results from susceptible mice model, this coronavirus mainly uses the olfactory route and neuron-to-neuron transmission to enter the CNS, and concentrates in the piriform cortex, the brain stem, and spinal cord (94). In immunocompetent adults, the pathogenicity of HCoV-OC43 are mainly attributed to the subsequent autoimmunity instead of viral replication, which undergoes a chronic and latent procedure for years, but in infants or immunocompromise individuals, this virus may lead to lethal acute encephalitis (14, 95, 96).

### Neuro-Invasive Property of SARS-CoV

In 2003, a previously uncharacterized coronavirus, SARS-CoV, was isolated from a cook in Guangdong, China. Different from the previously discovered HCoV-229E and HCoV-OC43, this type of coronavirus was not confined in the upper respiratory tracts, but lead to progressed pneumonia, which resulted in reduced alveolar diffuse function and the subsequent acute respiratory distress syndrome (ARDS). This coronavirus soon induced a global pandemic, and involved in 8,096 patients and 774 deaths (97, 98). Given the distinct syndromes compared with common viral pneumonia, this pandemic was finally named Severe Acute Respiratory Syndrome (SARS), and thus the pathogen was named SARS-CoV. SARS-CoV is a zoonotic virus originating from bats, and infected the palm civet as the second carrier, then finally managed to get across the species barrier to human via nosocomial transition and resulted in lethal pneumonia (99). The outbreak of SARS-CoV for the first time demonstrated the lethal pathogenicity of coronavirus, and the prevalence of coronavirus reservoirs in bats.

During SARS infection, an important complication is the multiple organ failure, usually observed in severe infection cases (100). This systemic damage of SARS used to be simply regarded as a result of immune dysregulation such as systemic inflammatory response syndrome (SIRS), until viral replication was detected in gastrointestinal, kidney, immune cells, and even brain, demonstrating that the SARS-CoV also produced direct toxicity to the organs by local viral replication (101). According to the reported autopsy results, SARS in brain tissue mainly confined to hypothalamus and cortex, leading to edema and degeneration of neurons. Indeed, the SARS central nervous infection presented a strong correlation with mortality risk, as the SARS was detected in all of the brain tissue autopsies. Another research found necrosis of neurons and hyperplasia of gliocytes in brain tissue specimen collected from SARS patient with the symptoms of significant central nervous infection, and anti-viral immune response was verified by increased immune cells and elevated cytokines level (102). Experimental data from hACE2 transgenic mice revealed that intranasal inoculation of SARS induced a delayed central nervous system infection, conforming to the process of secondary infection. Different from the regional distribution of SARS-CoV in brain specimens from clinical cases, once getting across the brain-blood barrier, the virus spread throughout the brain tissue of transgenic mice via the connection of neurons at a high effiency, especially in the regions of cortex, basal ganglia, and midbrain. Olfactory nerve retrograde and hematogenous routes were both suspected to account for the spread from respiratory tract to central nervous system, as virions were detected in blood and olfactory nerve (103, 104). Considering the brain infection presented a high correlation to death, it may exert neurogenic contribution to the respiratory failure, clinically the most common cause of death for SARS victims. Besides, the neuro-invasion and neuro-toxicity of SARS-CoV can also lead to chronic diseases and disorders even years after recovery from pneumonia. A series of somatic and psychologic symptoms relating to central nervous system prevailed among the survivor of SARS, including fatigue, sleep physiological changes, sleep disordered breathing and musculoskeletal pain (105). Sometimes the lesion can be latent and uncommon, such as a case of SARS-CoV induced olfactory dysfunction, which is rarely reported in typical peripheral neuropathy and leads to permanent anosmia (106). It needs to be pointed out that SARS-CoV shared the same receptor, ACE2, with SARS-CoV-2, and are genetically identical to the SARS-CoV-2, which means that these two viruses may undergo similar pathogenicity progress.

### Neuro-Invasive Property of SARS-CoV-2

SARS-CoV-2 shares a number of similarities to SARS-CoV, according to the clinical symptoms, viral sequence, infectivity, viral receptor, and possibly the neuro-invasive property. So far, SARS-CoV-2 has been found to cause extra-pulmonary infection, such as kidney, gastrointestinal and possibly heart (107–109). For neuro-invasive property of SARS-CoV-2, there are some cases about the neurological damage in COVID-19 patients, such as olfactory disorder and ocular abnormalities, both of which are related to peripheral nervous system disfunction, with no direct proves manifesting the viral replication in these tissue by RT-PCR (110). As for central nervous system, in the reports conducted by Chen et al., encephalopathy was seen in 23 of the 113 deceased patients, in which the patients presented anorexia, myalgia, and disorders of consciousness (111). However, there is still a lack of evidence from autopsy confirming the viral replication in brain tissue or cerebrospinal fluid, so it remains unclear whether these neurologic symptoms are led by viral infection or simply as a result of pulmonary encephalopathy. To confirm the existence of SARS-CoV-2 in central nervous system, more convincing proves from autopsy histologic examination is needed, especially the lesions in the olfactory bulb, rhinencephalon, paraventricular nucleus, and brain stem.

## Conclusion

In this review, we have summarized the possible mechanism for SARS-CoV-2 induced CNS infection, and conduct analysis on the potential of SARS-CoV-2 to cause CNS infection. At present the neuro-invasive property of SARS-CoV-2 are still unclear, but it seems that its infectivity in CNS is not as common as the previous SARS-CoV. It is no doubt that the central nervous system serves as an ideal target for SARS-CoV-2, with necessary receptor for viral tropism and feasible pathway for viral spread. At present, whether the SARS-CoV-2 can induce acute or chronic damage to CNS system, whether the SARS-CoV-2 can remain long-term latent infection in CNS system, and whether the latent SARS-CoV-2 will revive under given situation are all remaining to be explored in the follow-up visit. Considering the large number of patients involved, which is far more than the victims of SARS and MERS, clinicians should pay attention to the neurologic symptoms in COVID-19 patient, and act on an early stage. It should be pointed out for clinicians that the clinical manifestation of SARS-CoV-2 CNS infection could be staged, with possible existence of encephalitis in the acute phase (112), postinfectious symptoms in the subacute phase (113), and even involvement in patients vulnerable to neurodegenerative diseases in the chronic phase (114).

## Author Contributions

LZ conjointly conceptualized the idea for the review. JH and MZ performed the literature search, analyzed cited studies, and wrote the article. XT, YC, and AT critically revised the work and made changes and additions to its intellectual content. The corresponding author attests that all listed authors meet authorship criteria and that no others meeting the criteria have been omitted.

## Conflict of Interest

The authors declare that the research was conducted in the absence of any commercial or financial relationships that could be construed as a potential conflict of interest.
